# Association between bone mineral density and brain parenchymal atrophy and ventricular enlargement in healthy individuals

**DOI:** 10.18632/aging.102316

**Published:** 2019-09-30

**Authors:** In-Suk Bae, Jae Min Kim, Jin Hwan Cheong, Je Il Ryu, Myung-Hoon Han

**Affiliations:** 1 Department of Neurosurgery, Hanyang University Guri Hospital, Guri, Gyonggi-do, Korea

**Keywords:** cerebral atrophy, ventricular enlargement, osteoporosis, bone mineral density

## Abstract

Bone, vascular smooth muscle, and arachnoid trabeculae are composed of the same type of collagen. However, no studies have investigated the relationship between bone mineral density deterioration and cerebral atrophy, both of which occur in normal, healthy aging. Accordingly, we evaluated whether bone mineral density was associated with brain parenchymal atrophy and ventricular enlargement in healthy individuals. Intracranial cavity, brain parenchyma, and lateral ventricles volumes were measured using brain magnetic resonance imaging (MRI) with a semiautomated tool. We included 267 individuals with no history of dementia or other neurological diseases, who underwent one or more dual-energy X-ray absorptiometry scans and brain MRIs simultaneously (within 3 years of each other) at our hospital over an 11-year period. We found that progression of brain parenchymal atrophy was positively associated with bone mineral density after full adjustment (B, 0.94; P < 0.001). In addition, individuals with osteoporosis showed more parenchymal atrophy among those younger than 80 years. In addition, we observed greater ventricular enlargement in individuals with osteoporosis among those older than 80 years. We believe that osteoporosis may play a role in the acceleration of parenchymal atrophy during the early-stages, and ventricular enlargement in the late-stages, of normal aging-related cerebral atrophy.

## INTRODUCTION

Cerebral atrophy and ventricular enlargement which occur during the normal aging process are known to lead to dementia, memory disorders, and cognitive impairment [1]. Physiological aging of the brain is at an increased risk of dementia, and addressing this public health problem is a major challenge currently facing medical research [2].

Bone, blood vessels, and arachnoid trabeculae share the same collagen type (type 1 collagen) [3–5]. Osteogenesis imperfecta, a disease caused by mutations in genes encoding type 1 collagen, affects the cardio- or cerebrovascular and ventricular systems, as well as bone [6–9]. Osteoporosis is a systemic disease that is strongly associated with genes that encode for the components of type 1 collagen [10, 11].

To the best of our knowledge, there are no studies investigating the association between bone mineral density (BMD) and cerebral atrophy in healthy individuals. As bone, vascular smooth muscle, and arachnoid trabeculae are composed of the same type of collagen, we hypothesized that osteoporosis may be linked to brain parenchymal atrophy and ventricular enlargement in the process of normal aging-related cerebral atrophy. Therefore, we investigated this notion by analyzing T-scores from dual-energy X-ray absorptiometry (DXA) and measuring the intracranial cavity volume (ICV), brain parenchymal volume (BPV), and lateral ventricles volume (LVV) from brain magnetic resonance imaging (MRI) using a 3D slicer software in healthy individuals.

## RESULTS

### Characteristics of study individuals

The average age of the subjects was 67.4 years, and 81.3% of individuals were women. A total of 72 individuals had osteoporosis and the overall mean T-score was −1.6. The mean ICV, BPV, and LVV were 1224.9 cc, 1026.4 cc, and 29.3 cc, respectively. The mean volume percentages of the brain parenchyma and lateral ventricles to the intracranial cavity were 83.8% and 2.4%, respectively. We found significant differences in ICV, BPV, LVV, and the intracranial cavity volume percentages of the brain parenchyma ((BPV/ ICV) × 100(%)) and lateral ventricles ((LVV/ICV) × 100(%)) between subjects with osteoporosis and those without. Further descriptive data are shown in Table 1.

**Table 1:** Characteristics of the study individuals according to osteoporosis.

**Characteristics**	**Osteoporosis (-)**	**Osteoporosis (+)**	**Total**	**P**
Number	195	72	267	
Sex, female, n (%)	159 (81.5)	58 (80.6)	217 (81.3)	0.995
Age at the time of brain MRI, mean ± SD, y	65.4 ± 12.3	72.7 ± 9.3	67.4 ± 12.0	<0.001
Time interval between brain MRI and DXA, median (IQR), days	193.0 (35.0–486.0)	154.5 (22.0–363.5)	184.0 (33.0–441.0)	0.077
ICV, mean ± SD, cc	1232.7 ± 90.7	1203.9 ± 89.9	1224.9 ± 91.2	0.021
BPV, mean ± SD, cc	1042.1 ± 98.8	983.8 ± 79.5	1026.4 ± 97.3	< 0.001
LVV, mean ± SD, cc	27.9 ± 14.2	33.1 ± 16.4	29.3 ± 15.0	0.011
Volume percentage of the brain parenchyma ((BPV/ICV)×100), mean ± SD, %	84.5 ± 4.7	81.8 ± 3.9	83.8 ± 4.6	< 0.001
Volume percentage of the lateral ventricles ((LVV/ICV)×100), mean ± SD, %	2.3 ± 1.1	2.8 ± 1.4	2.4 ± 1.2	0.007
T-score, mean ± SD	-1.1 ± 0.9	-3.0 ± 0.5	-1.6 ± 1.2	< 0.001
Lumbar spine	-0.7 ± 1.2	-2.8 ± 0.7	-1.3 ± 1.5	< 0.001
Femur neck	-0.6 ± 1.2	-2.1 ± 1.0	-1.0 ± 1.3	< 0.001
BMI, mean ± SD, kg/m^2^	24.5 ± 3.5	22.5 ± 3.5	24.0 ± 3.6	< 0.001
Height, mean ± SD, cm	156.4 ± 8.0	152.8 ± 7.9	155.4 ± 8.1	0.001
Weight, mean ± SD, kg	59.9 ± 9.6	52.5 ± 8.3	57.9 ± 9.8	< 0.001
Hypertension, n (%)	91 (46.7)	39 (54.2)	130 (48.7)	0.342
Diabetes, n (%)	68 (34.9)	19 (26.4)	87 (32.6)	0.244
Alcohol, n (%)	27 (13.8)	7 (9.7)	34 (12.7)	0.490
Smoking, n (%)	14 (7.2)	9 (12.5)	23 (8.6)	0.259

SD: standard deviation; MRI: magnetic resonance imaging; DXA: dual-energy X-ray absorptiometry; IQR: interquartile range; ICV: intracranial cavity volume; BPV: brain parenchymal volume; LVV: lateral ventricles volume; BMI: body mass index.

Detailed information on the bone densitometric parameters including bone mineral content (BMC), BMD, T-, and Z-score in the study participants classified by age group are also presented in Table 2. In addition, we assessed the relationships between volume percentage of brain parenchyma and lateral ventricles and different bone densitometric parameters using Pearson correlation analysis with/without adjustment for age (Tables 3 and 4).

**Table 2:** Descriptive statistics of bone densitometric parameters in the study participants classified by age group.

**Bone densitometric parameters**	**< 65 y (n=95)**	**65 y–79 y (n=131)**	**80 y or older (n=41)**	**Total (n=267)**	**P**
Femur neck T-score, mean ± SD	-0.35 ± 1.24	-1.16 ± 1.15	-1.96 ± 1.18	-1.00 ± 1.31	< 0.001
Lumbar spine T-score, mean ± SD	-0.94 ± 1.42	-1.41 ± 1.48	-1.79 ± 1.25	-1.30 ± 1.45	0.003
T-score (lower value between the lumbar spine and femoral neck), mean ± SD	-1.14 ± 1.24	-1.76 ± 1.10	-2.31 ± 1.01	-1.63 ± 1.21	< 0.001
Femur neck BMC, mean ± SD, g	28.76 ± 6.66	26.74 ± 7.80	23.82 ± 8.32	27.01 ± 7.65	0.002
Lumbar spine BMC, mean ± SD, g	54.63 ± 14.22	48.71 ± 17.70	45.33 ± 15.88	50.30 ± 16.56	0.003
Femur neck BMD, mean ± SD, g/cm^2^	0.83 ± 0.14	0.74 ± 0.13	0.64 ± 0.15	0.75 ± 0.15	< 0.001
Lumbar spine BMD, mean ± SD, g/cm^2^	0.90 ± 0.16	0.85 ± 0.18	0.80 ± 0.15	0.86 ± 0.17	0.004
Femur neck Z-score, mean ± SD	0.26 ± 1.22	0.20 ± 0.96	-0.11 ± 1.12	0.17 ± 1.09	0.191
Lumbar spine Z-score, mean ± SD	0.13 ± 1.16	0.54 ± 1.07	0.51 ± 0.93	0.39 ± 1.10	0.014

SD: standard deviation; BMC: bone mineral content; BMD: bone mineral density.

**Table 3:** Pearson correlation coefficients among the volume percentage of brain parenchyma and lateral ventricles to the intracranial cavity and bone densitometric parameters of the study cohort.

	**Volume percentage of the brain parenchyma ((BPV/ICV) × 100%)**	**Volume percentage of the lateral ventricles ((LVV/ICV) × 100%)**	**Femur neck T-score**	**Lumbar spine T-score**	**T-score (lower value between the lumbar spine and femoral neck)**	**Femur neck BMC**	**Lumbar spine BMC**	**Femur neck BMD**	**Lumbar spine BMD**	**Femur neck Z-score**	**Lumbar spine Z-score**
Volume percentage of the brain parenchyma ((BPV/ICV) × 100%)	1	-0.418^**^	0.261^**^	0.321^**^	0.365^**^	0.131^*^	0.193^**^	0.262^**^	0.310^**^	0.115	0.122^*^
Volume percentage of the lateral ventricles ((LVV/ICV) × 100%)		1	-0.254^**^	-0.151^*^	-0.219^**^	-0.080	-0.062	-0.242^**^	-0.144^*^	-0.115	-0.017
Femur neck T-score			1	0.594^**^	0.782^**^	0.615^**^	0.499^**^	0.906^**^	0.593^**^	0.816^**^	0.408^**^
Lumbar spine T-score				1	0.888^**^	0.481^**^	0.764^**^	0.621^**^	0.987^**^	0.538^**^	0.829^**^
T-score (lower value between the lumbar spine and femoral neck)					1	0.574^**^	0.703^**^	0.808^**^	0.882^**^	0.710^**^	0.692^**^
Femur neck BMC						1	0.624^**^	0.767^**^	0.504^**^	0.563^**^	0.218^**^
Lumbar spine BMC							1	0.592^**^	0.780^**^	0.382^**^	0.484^**^
Femur neck BMD								1	0.632^**^	0.843^**^	0.382^**^
Lumbar spine BMD									1	0.537^**^	0.825^**^
Femur neck Z-score										1	0.589^**^
Lumbar spine Z-score											1

ICV: intracranial cavity volume; BPV: brain parenchymal volume; LVV: lateral ventricles volume; BMC: bone mineral content;

BMD: bone mineral density ^*^P<0.05 ^**^P<0.01

**Table 4:** Age-adjusted Pearson correlation coefficients among the volume percentage of brain parenchyma and lateral ventricles to the intracranial cavity and bone densitometric parameters of the study cohort.

	**Volume percentage of the brain parenchyma ((BPV/ICV) × 100%)**	**Volume percentage of the lateral ventricles ((LVV/ICV) × 100%)**	**Femur neck T-score**	**Lumbar spine T-score**	**T-score (lower value between the lumbar spine and femoral neck)**	**Femur neck BMC**	**Lumbar spine BMC**	**Femur neck BMD**	**Lumbar spine BMD**	**Femur neck Z-score**	**Lumbar spine Z-score**
Volume percentage of the brain parenchyma ((BPV/ICV) × 100%)	1	-0.251^**^	0.075	0.249^**^	0.233^**^	0.048	0.104	0.081	0.239^**^	0.099	0.245^**^
Volume percentage of the lateral ventricles ((LVV/ICV) × 100%)		1	-0.066	-0.051	-0.054	0.012	0.048	-0.056	-0.045	-0.099	-0.123^*^
Femur neck T-score			1	0.564^**^	0.743^**^	0.602^**^	0.459^**^	0.886^**^	0.565^**^	0.877^**^	0.551^**^
Lumbar spine T-score				1	0.888^**^	0.458^**^	0.752^**^	0.594^**^	0.986^**^	0.540^**^	0.912^**^
T-score (lower value between the lumbar spine and femoral neck)					1	0.552^**^	0.686^**^	0.774^**^	0.883^**^	0.741^**^	0.834^**^
Femur neck BMC						1	0.608^**^	0.770^**^	0.482^**^	0.563^**^	0.264^**^
Lumbar spine BMC							1	0.564^**^	0.769^**^	0.379^**^	0.549^**^
Femur neck BMD								1	0.608^**^	0.902^**^	0.517^**^
Lumbar spine BMD									1	0.538^**^	0.905^**^
Femur neck Z-score										1	0.612^**^
Lumbar spine Z-score											1

ICV: intracranial cavity volume; BPV: brain parenchymal volume; LVV: lateral ventricles volume; BMC: bone mineral content.

BMD: bone mineral density ^*^P<0.05 ^**^P<0.01.

### Association between T-score and volume percentages of the brain parenchyma and lateral ventricles to the intracranial cavity

**Figure 1:**
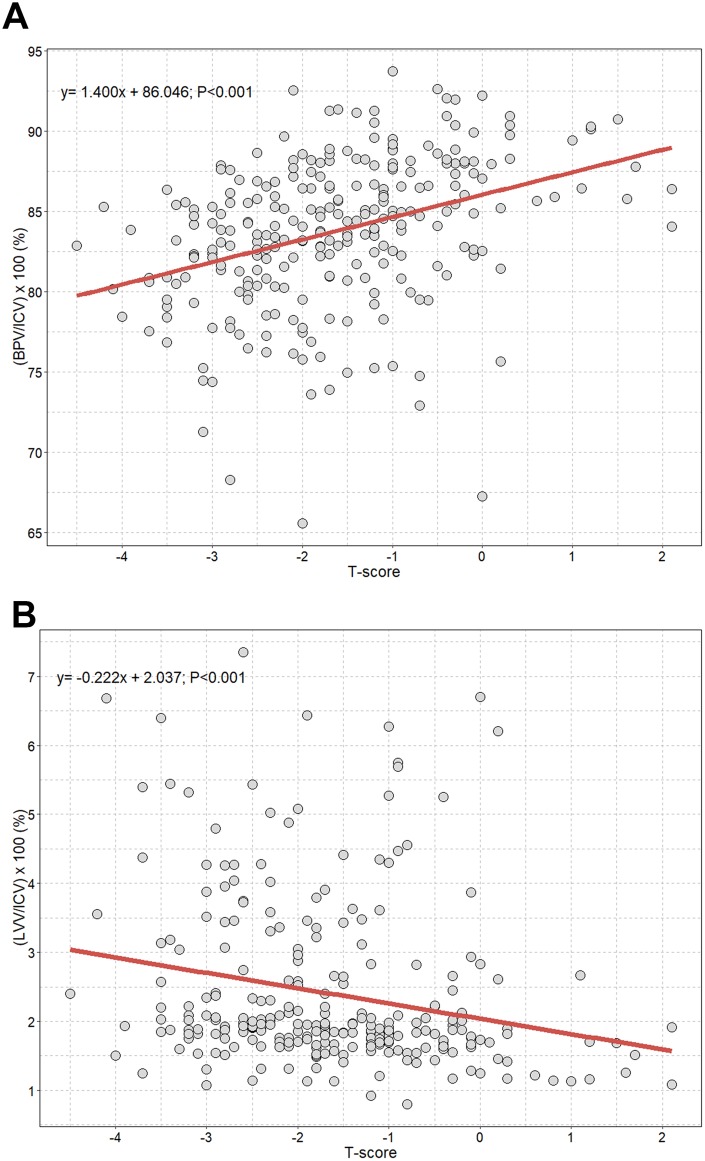
**Scatterplot with linear regression line showing the association between T-score and volume percentages of brain parenchyma and lateral ventricles.** (**A**) Volume percentage of brain parenchyma to intracranial cavity; (**B**) volume percentage of lateral ventricles to intracranial cavity. ICV=intracranial cavity volume: BPV=brain parenchymal volume; LVV=lateral ventricles volume.

Figure 1 shows significant positive and negative correlations between T-scores and the intracranial cavity volume percentages of the brain parenchyma and lateral ventricles. We observed a statistically significant increase in the intracranial cavity volume percentage of the brain parenchyma ((BPV/ICV)×100(%)) with increasing T-scores (B, 1.400; P<0.001) (Figure 1A). Conversely, T-scores were negatively associated with intracranial cavity volume percentages of the lateral ventricles ((LVV/ICV)×100(%)) (B, −0.222; P<0.001) (Figure 1B). After adjusting for all covariates including age, we observed an independent positive correlation between T-scores and intracranial cavity volume percentages of the brain parenchyma, with an approximate increase of 0.9 % per 1 T-score increase (B, 0.94; 95% CI, 0.48 to 1.40; P<0.001; Table 5). However, percentage of lateral ventricles showed no significant association with T-score in the multivariable linear analysis (B, −0.06; 95% CI, −0.18 to 0.06; P=0.339).

**Table 5:** Multivariable linear regression analysis of the volume percentage of brain parenchyma and lateral ventricles to the intracranial cavity according to several clinical factors of the study cohort.

	**Multivariable linear regression analysis**			
	**Volume percentage of the brain parenchyma ((BPV/ICV)×100%)**		**Volume percentage of the lateral ventricles ((LVV/ICV)×100%)**	
Variable	β (95% CI)	P value	β (95% CI)	P value
Intercept	96.05		0.58	
Male (vs female)	-0.57 (-1.95 to 0.80)	0.412	0.40 (0.03 to 0.76)	0.033
Age at the time of brain MRI (per 1-year increase)	-0.15 (-0.20 to -0.10)	<0.001	0.04 (0.03 to 0.05)	<0.001
Time interval between brain MRI and DXA (per 1-month increase)	-0.01 (-0.06 to 0.04)	0.667	0.01 (-0.01 to 0.02)	0.291
T-score	0.94 (0.48 to 1.40)	<0.001	-0.06 (-0.18 to 0.06)	0.339
BMI (per 1 BMI increase)	-0.02 (-0.16 to 0.13)	0.840	-0.05 (-0.09 to -0.02)	0.007
Hypertension	0.53 (-0.59 to 1.65)	0.352	0.13 (-0.17 to 0.43)	0.397
Diabetes	-1.41 (-2.47 to -0.35)	0.009	0.17 (-0.11 to 0.45)	0.243
Alcohol	0.09 (-1.44 to 1.62)	0.910	0.16 (-0.24 to 0.57)	0.429
Smoking	0.33 (-1.53 to 2.19)	0.728	-0.43 (-0.93 to 0.07)	0.089

ICV: intracranial cavity volume; BPV: brain parenchymal volume; LVV: lateral ventricles volume; CI: confidence interval; MRI: magnetic resonance imaging; DXA: dual-energy X-ray absorptiometry; BMI: body mass index.

### Other factors associated with volume percentages of the brain parenchyma and lateral ventricles

As expected, age was independently associated with volume percentages of the brain parenchyma and lateral ventricles (B, −0.15; 95% CI, −0.20 to −0.10; P<0.001; B, 0.04; 95% CI, 0.03 to 0.05; P<0.001, respectively; Table 5). Diabetes was an independent predictor of lower volume percentages of the brain parenchyma (B, −1.41; 95% CI, −2.47 to −0.35; P=0.009). In addition, we observed a statistically significant decrease in the volume percentage of lateral ventricles with an increasing body mass index (BMI) (B, −0.05; 95% CI, −0.09 to −0.02; P=0.007). We additionally investigated the association between T-score and BMI in the study participants (Supplementary Figure 1).

### Comparison of volume percentage of brain parenchyma and lateral ventricles between age groups according to osteoporosis

**Figure 2:**
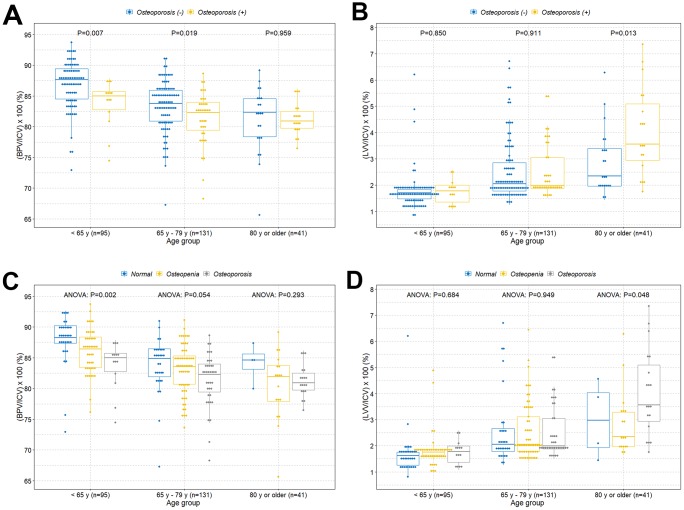
**Boxplots with dot plots of the volume percentages of brain parenchyma and lateral ventricles classified by age groups according to two different BMD groups.** (**A**) Volume percentage of brain parenchyma to intracranial cavity classified by age groups according to osteoporosis; (**B**) volume percentage of lateral ventricles to intracranial cavity classified by age groups according to osteoporosis; (**C**) volume percentage of brain parenchyma to intracranial cavity classified by age groups based on normal, osteopenia, and osteoporosis groups; (**D**) volume percentage of lateral ventricles to intracranial cavity classified by age groups based on normal, osteopenia, and osteoporosis groups. BMD=bone mineral density; ICV=intracranial cavity volume; BPV=brain parenchymal volume; LVV=lateral ventricles volume.

**Figure 3:**
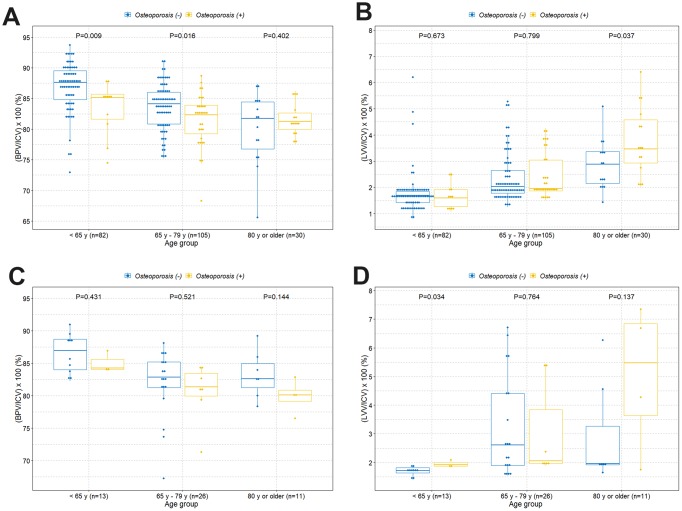
**Boxplots with dot plots of the volume percentages of brain parenchyma and lateral ventricles classified by age groups according to osteoporosis based on sex group.** (**A**) Volume percentage of brain parenchyma to intracranial cavity in females; (**B**) volume percentage of lateral ventricles to intracranial cavity in females; (**C**) volume percentage of brain parenchyma to intracranial cavity in males; (**D**) volume percentage of lateral ventricles to intracranial cavity in males. ICV=intracranial cavity volume; BPV=brain parenchymal volume; LVV=lateral ventricles volume.

**Figure 4:**
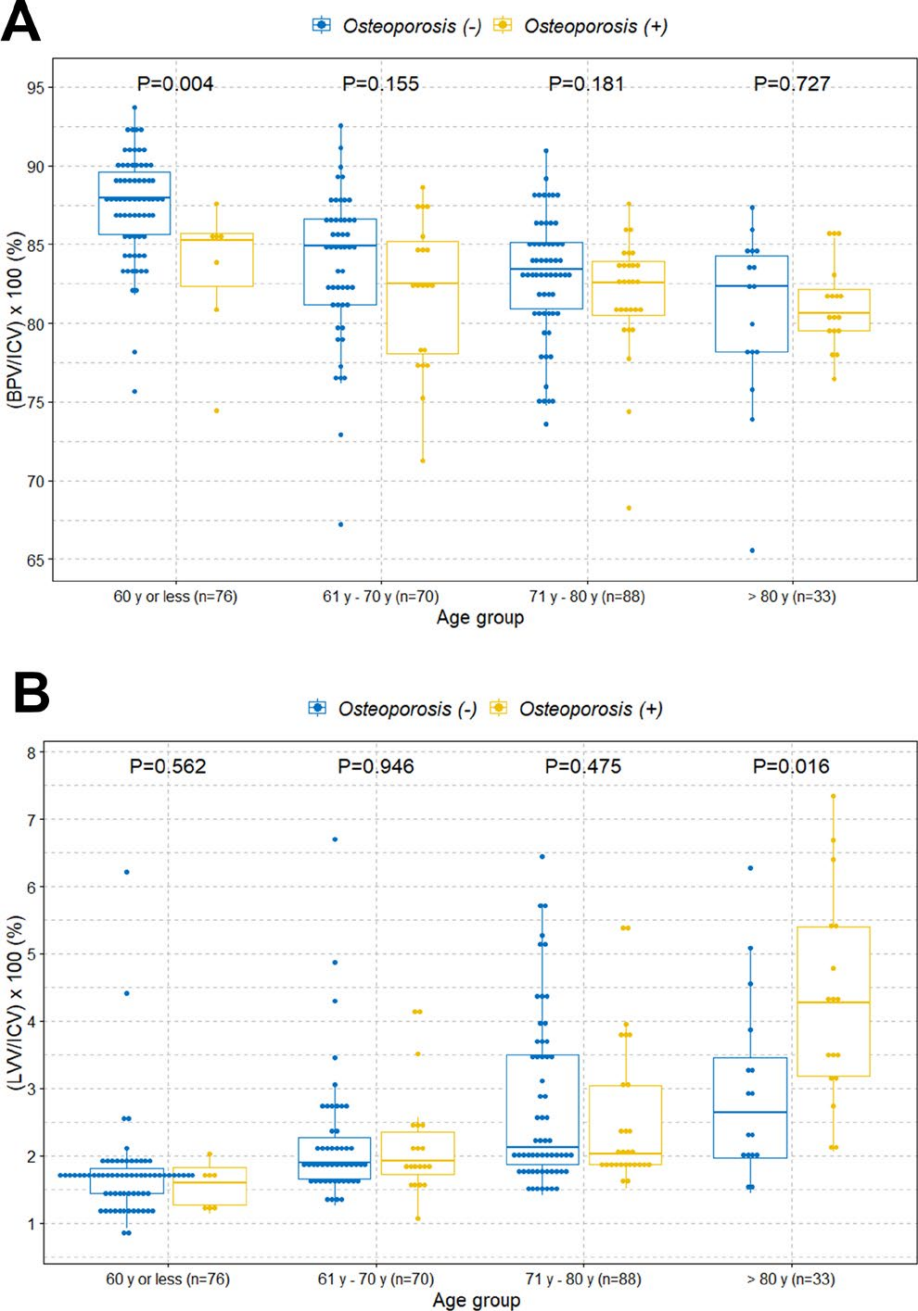
**Boxplots with dot plots of the volume percentages of brain parenchyma and lateral ventricles classified by different age groups according to osteoporosis.** (**A**) Volume percentage of brain parenchyma to intracranial cavity; (**B**) volume percentage of lateral ventricles to intracranial cavity. ICV=intracranial cavity volume; BPV=brain parenchymal volume; LVV=lateral ventricles volume.

The boxplot shows a statistically significant tendency of lower intracranial cavity volume percentage of the brain parenchyma in individuals with osteoporosis among those under 80 years of age (Figure 2A). However, there was no difference of volume percentage of brain parenchyma between individuals with and without osteoporosis in individuals older than 80 years. Surprisingly, we found a significant higher volume percentage of lateral ventricles in individuals with osteoporosis among those older than 80 years (Figure 2B). However, there were no significant differences of volume percentage of lateral ventricles between individuals with and without osteoporosis among those under 80 years. We also observed similar tendencies in associations when the study participants were categorized into normal, osteopenia, and osteoporosis groups (Figure 2C and 2D). We further evaluated those relationships based on sex and age groups. Similar tendencies in associations were observed in the female group (Figure 3A and 3B). However, male participants showed no significant differences in volume percentage of brain parenchyma and lateral ventricles between individuals with and without osteoporosis, which is thought to be due to a low sample size (Figure 3C and 3D). When study participants were divided into different age groups, we observed an overall tendency towards a lower volume percentage of brain parenchyma in individuals with osteoporosis among those under 80 years of age and higher volume percentage of lateral ventricles in individuals with osteoporosis among those older than 80 years (Figure 4).

Table 6 shows the characteristics of the study participants classified by age group. We observed a significant decrease in T-score with a higher age group. The mean volume percentages of the brain parenchyma in age < 65 years, 65–79 years, and 80 years or older were 86.4%, 82.7%, and 81.1%, respectively. In addition, the mean volume percentage of the lateral ventricles according to the same age groups were 1.8%, 2.5%, and 3.4%, respectively. There were no significant differences in other clinical factors between age groups, except for hypertension.

**Table 6:** Characteristics of the study individuals classified by age group.

**Characteristics**	**< 65 y**	**65 y–79 y**	**80 y or older**	**Total**	**P**
Number	95	131	41	267	
Sex, female, n (%)	82 (86.3)	105 (80.2)	30 (73.2)	217 (81.3)	0.177
Time interval between brain MRI and DXA, median (IQR), days	193.0 (20.0–577.0)	168.0 (40.0–473.0)	191.0 (26.5–321.5)	184.0 (33.0–441.0)	0.315
ICV, mean ± SD, cc	1240.5 ± 83.3	1221.0 ± 94.4	1201.5 ± 94.2	1224.9 ± 91.2	0.016
BPV, mean ± SD, cc	1071.0 ± 82.9	1010.0 ± 94.6	975.3 ± 97.4	1026.4 ± 97.3	< 0.001
LVV, mean ± SD, cc	22.1 ± 10.0	31.0 ± 14.7	40.3 ± 17.1	29.3 ± 15.0	< 0.001
(BPV/ICV)×100, mean ± SD, %	86.4 ± 4.0	82.7 ± 4.3	81.1 ± 4.2	83.8 ± 4.6	< 0.001
(LVV/ICV)×100, mean ± SD, %	1.8 ± 0.7	2.5 ± 1.2	3.4 ± 1.5	2.4 ± 1.2	< 0.001
(BPV/ICV)×100, median (IQR), %	87.1 (84.3–88.8)	83.2 (80.5–85.4)	81.4 (78.8–84.1)	84.2 (81.0–87.1)	< 0.001
(LVV/ICV)×100, median (IQR), %	1.7 (1.4–1.9)	2.0 (1.8–3.0)	3.2 (2.0–4.3)	1.9 (1.7–2.7)	< 0.001
T-score, mean ± SD	-1.1 ± 1.2	-1.8 ± 1.1	-2.3 ± 1.0	-1.6 ± 1.2	< 0.001
BMI, mean ± SD, kg/m^2^	23.7 ± 3.5	24.8 ± 3.7	22.1 ± 2.7	24.0 ± 3.6	0.233
Hypertension, n (%)	21 (22.1)	79 (60.3)	30 (73.2)	130 (48.7)	< 0.001
Diabetes, n (%)	26 (27.4)	48 (36.6)	13 (31.7)	87 (32.6)	0.337
Alcohol, n (%)	18 (18.9)	11 (8.4)	5 (12.2)	34 (12.7)	0.063
Smoking, n (%)	11 (11.6)	9 (6.9)	3 (7.3)	23 (8.6)	0.437

SD: standard deviation; MRI: magnetic resonance imaging; DXA: dual-energy X-ray absorptiometry; IQR: interquartile range; ICV: intracranial cavity volume; BPV: brain parenchymal volume; LVV: lateral ventricles volume; BMI: body mass index.

### Comparison of volume percentage of brain parenchyma and lateral ventricles according to medical history

Study participants were classified according to their medical conditions (smoking history was not included in the analysis due to low sample size) (Supplementary Figures 2–4). We found an overall tendency towards lower volume percentage of brain parenchyma and higher volume percentage of lateral ventricles in individuals with hypertension and diabetes. However, more significant differences in volume percentage of brain parenchyma and lateral ventricles were observed between individuals with and without osteoporosis in participants without hypertension and diabetes.

## DISCUSSION

We found that the progression of brain parenchymal atrophy was positively associated with bone mineral density after adjusting for covariates, including age, in healthy individuals. In addition, individuals with osteoporosis showed significantly lower intracranial cavity volume percentages of brain parenchyma among those under 80 years of age. On the contrary, we observed a significantly higher volume percentage of lateral ventricles in individuals with osteoporosis older than 80 years. To our knowledge, this study is the first to suggest that bone mineral density is related to brain parenchymal atrophy and ventricular enlargement in healthy individuals.

It is well known that brain atrophy and ventricular dilatation accelerate with increasing age [12]. The prevalence of dementias such as Alzheimer’s disease increases exponentially after the age of 65 years [13]. A previous study of autopsy cases found that brain atrophy was detected in both normal individuals and patients with Alzheimer's-type dementia over 70 years of age [14]. However, the investigators observed that brain atrophy was accompanied by ventricular dilatation in all Alzheimer's-type dementia patients, but not in healthy individuals. Therefore, the authors concluded that brain atrophy may persist with increasing age whereas ventricular dilatation likely does not progress in healthy individuals; conversely, rapidly progressive brain atrophy with ventricular dilatation is likely a common characteristic of Alzheimer's-type dementia in patients over 70 years of age. A previous study revealed that patients with Alzheimer's disease had greater ventricular enlargement than subjects with mild cognitive impairment and normal elderly controls [15]. The authors suggested that ventricular enlargement represents a feasible short-term marker of disease progression in subjects with mild cognitive impairment and in those with Alzheimer's disease. Both normal aging and Alzheimer’s disease are associated with changes in grey and white matter volume and white matter integrity, and it is hypothesized that Alzheimer’s disease might simply reflect an accelerated aging process [16]; this makes, the separation between normal aging and Alzheimer's disease is frequently challenging. Accordingly, we excluded patients with a history of Alzheimer’s disease and other types of dementia to investigate the relationships between bone mineral density and cerebral atrophy in healthy individuals.

According to the studies mentioned previously, we hypothesized that normal aging may lead firstly to brain parenchymal atrophy and then ventricular dilatation later in normal aging-related cerebral atrophy. In addition, according to our findings, we also postulate that osteoporosis may have a role in the acceleration of parenchymal atrophy (cortical atrophy) in the early-stages of normal aging-related cerebral atrophy and ventricular enlargement (subcortical atrophy) in the late-stages. Similar to our findings, a previous study reported that BMD is reduced in the earliest clinical stages of Alzheimer's disease in both men and women [17].

Osteoporosis is a systemic disease that affects bone mass and microarchitecture throughout the body [10]. Moreover, osteoporosis is strongly associated with genetic components of type 1 collagen such as *COL1A1* and *COL1A2* [11]. Polymorphisms in COL1A1 can result in low bone density properties in individuals in early puberty, indicating that it may have potential physiological effects on bone turnover and collagen metabolism [18]. It is well documented that type 1 collagen is a major component of bone; moreover, skin, tendons, vascular smooth muscle, and other organs are also composed of type 1 collagen to varying extents. A previous study revealed that morphometric changes in the cortical capillary network occur in Alzheimer’s disease in a region-specific manner, and that this phenomenon may be related to cortical atrophy in the affected regions [19]. Cerebral microvascular abnormalities have been reported as playing a role in the pathogenesis of Alzheimer’s disease [20–24]. Previous studies have consistently found that a low BMD correlates with increased arterial stiffness and atherosclerosis, especially in postmenopausal women, who are more vulnerable to osteoporosis [25–28]. Therefore, we hypothesized that an osteoporotic condition with a strong genetic component (i.e., dysregulated type 1 collagen genes) and leads to systemic disease may also cause the degeneration of vascular smooth muscle cells, including the cortical capillary network that is also composed of type 1 collagen.

In addition, both the arachnoid trabeculae and granulations are composed of type 1 collagen, which is also the principal component of the bone matrix protein [3]. The arachnoid trabeculae is composed of abundant strands of collagen tissue that connects and brings stability to the subarachnoid space and cerebrospinal fluid flow [29]. Osteogenesis imperfecta, which is caused by mutations in the type 1 procollagen genes (COL1A1/COL1A2), is associated with communicating hydrocephalus [6, 7]. We previously hypothesized that systemic osteoporosis may negatively affect the integrity of arachnoid trabeculae and granulation, which are also composed of type 1 collagen [30]. Therefore, we hypothesized that a greater weakening of the arachnoid trabeculae and granulation in osteoporotic individuals, may accelerate the ventricular enlargement in the late-stage of normal aging-related cerebral atrophy.

Consistent with a previous study, we also observed that diabetes was associated with brain parenchymal atrophy [31]. In addition, our findings showed that lower BMI was associated with cerebral ventricular enlargement. As our study also showed a positive association between T-score and BMI, it is generally accepted that BMI and bone mineral density are positively correlated [32, 33]. Therefore, we believe that BMI is more linearly negatively associated with ventricle volume in all age groups, whereas BMD is negatively associated with ventricle volume, especially in the older age group.

Our study has some limitations. First, because of the study's retrospective nature, the interval between undergoing DXA and brain MRI was not consistent between all participants. A previous study found that osteoporosis develops in less than 10% of older women (≥65 years) over BMD rescreening intervals of approximately 15 years, 5 years, and 1 year for women with normal bone density, moderate osteopenia, and advanced osteopenia, respectively [34]. On the basis of this finding of the relatively slow osteoporosis progression in older women, our maximum time interval of 3 years between DXA and brain MRI may be acceptable for analysis. Second, technical errors may have occurred during measurement of brain parenchyma and lateral ventricles volumes with the 3D slicer. Third, structural changes in the brain may affect skeletal system structure and functions. Previous studies reported on the possible role of the nervous system in bone mass control [35–38]. According to these studies, brain parenchymal atrophy and ventricular enlargement may affect bone remodelling and cause osteoporosis; this fact can affect our study results. However, this mechanism is hypothetical and further studies in humans are required. In addition, we believe that our study is valuable because, to the best of our knowledge, there have been no previous clinical studies investigating the association between BMD and brain atrophy and ventricle enlargement in the elderly. We assessed the quantitative volume measurements of intracranial cavity, brain parenchyma, and lateral ventricles of participants using 3D reconstructed brain MRI images. Lastly, we included only Korean individuals, which may limit the generalizability of the results. However, data accuracy owing to consistent environmental conditions is a strength of a single-center study.

In summary, we observed a significant linear association between BMD and brain parenchymal atrophy. We believe that osteoporosis may have a role in the acceleration of parenchymal atrophy in the early-stages of normal aging-related cerebral atrophy and ventricular enlargement in the late-stages of normal aging-related cerebral atrophy. We expect the findings of this study will expand our understanding of the association between osteoporosis and brain atrophy.

## MATERIALS AND METHODS

### Study design

**Figure 5:**
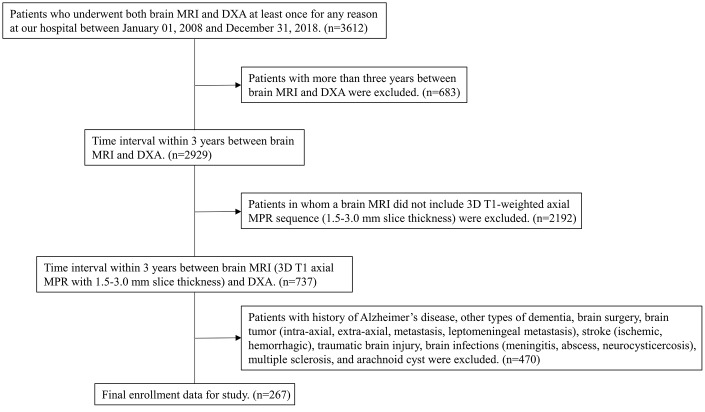
**Flow chart of the process for selecting eligible patients from individuals who underwent both brain MRI and DXA in our hospital during the period of January 1, 2008, to December 31, 2018.** MRI=magnetic resonance imaging; DXA=dual-energy X-ray absorptiometry; MPR=multiplanar reconstruction.

We retrospectively recruited individuals who underwent both brain MRI and DXA at least once for any reason in our hospital from January 1, 2008 to December 31, 2018. We initially identified 3,612 individuals whose records indicated one or more procedure codes for DXA and brain MRI simultaneously. In individuals who underwent DXA more than once, the lowest T-score from among the multiple DXA T-score values was considered. If multiple MRIs were performed, we considered that which was performed closest to the date of the DXA with the lowest T-score. We then excluded 3,345 individuals using stepwise methods for the following reasons: (1) more than 3 years elapsed between DXA and brain MRI (to reduce time heterogeneity) (n=683); (2) individuals in whom a brain MRI did not include T1-weighted axial multiplanar reconstruction (MPR) sequence (3-mm-thick slices) (n=2192); and (3) history of Alzheimer’s disease, other types of dementia, brain surgery, brain tumor (intra-axial, extra-axial, metastasis, leptomeningeal metastasis), stroke (ischemic, hemorrhagic), traumatic brain injury, brain infections (meningitis, abscess, neurocysticercosis), multiple sclerosis, and arachnoid cyst (n=470). Ultimately, we enrolled 267 individuals with no history of Alzheimer’s disease, other types of dementia, brain surgery, brain tumor, stroke, traumatic brain injury, brain infections, multiple sclerosis, or arachnoid cysts who underwent one or more DXA and brain MRI (included 3D T1-weighted multiplanar reconstruction (MPR) sequence), within 3 years of each other at our hospital over an 11-year period (Figure 5).

This study was approved by the Institutional Review Board of Hanyang University Guri Hospital, Korea, and conformed to the tenets of the Declaration of Helsinki. Owing to the retrospective nature of the study, the need for informed consent was waived. All individual records were anonymized prior to analysis.

### BMD measurement

DXA to assess the BMC (g) and BMD (g/cm^2^) of the lumbar spine L1–L4 and femoral neck was performed using a Discovery Wi DXA system (Hologic, Bedford, MA) in all study subjects. All testing was conducted by licensed technicians. The BMD values were converted into a T- and Z-score. T-score reference ranges were calculated using data from healthy young Asian women provided by the bone densitometry equipment manufacturer. Z-Scores were also calculated from the mean BMD of a healthy Asian population of the same age and sex. Study individuals were categorized as normal, osteopenia, or osteoporosis based on the World Health Organization T-score classification: osteoporosis was defined as a T-score (≤ −2.5), osteopenia as a T-score > −2.5 and ≤ −1.0), and normal BMD as a T-score > −1.0. The lower T-score value between the lumbar spine and femoral neck was adopted as the T-score for the study.

### Brain MRI image acquisition

All 3D T1-weighted MPR MR images (slice thicknesses, 1.5-3.0 mm) were obtained with an Ingenia 3.0 Tesla CX (Philips Ingenia, Philips Medical Systems, Böblingen, Germany) and Achieva 3.0 Tesla TX (Philips Achieva, Philips Medical Systems, Böblingen, Germany) scanners at our hospital. Technical parameters were standardized as following; TE: 4.61 ms; TR: 8.29 ms; field of view: 207 × 207 mm; matrix size: 224*224 pixels. A previous study described that MPR sequences with a 3.0 Tesla MRI provided excellent visualization for the inner structures of head and brain [39].

### Volumetric assessment of intracranial cavity, brain parenchyma, and lateral ventricles

We measured ICV, BPV, and LVV with a 3D slicer software in its latest version, 4.10.1. (http://www.slicer.org). The reliability of the 3D slicer has been described elsewhere, including detailed descriptions of the slicer’s various functions [40, 41]. All procedures were performed by a well-trained 3D-slicer user. The T1-weighted MR images were used for the analysis in all study individuals as T1-weighted MR imaging is appropriate for skull stripping (https://www.slicer.org/wiki/Modules:SkullStripperModule) and the measurement of brain parenchyma and CSF space volumes using a slicer [42]. In addition, as the T1 MPR sequence with the 3.0 Tesla MRI in our hospital showed a relatively thin slice thickness of 1.5–3.0 mm, we were able to perform more accurate volumetric measurements.

**Figure 6:**
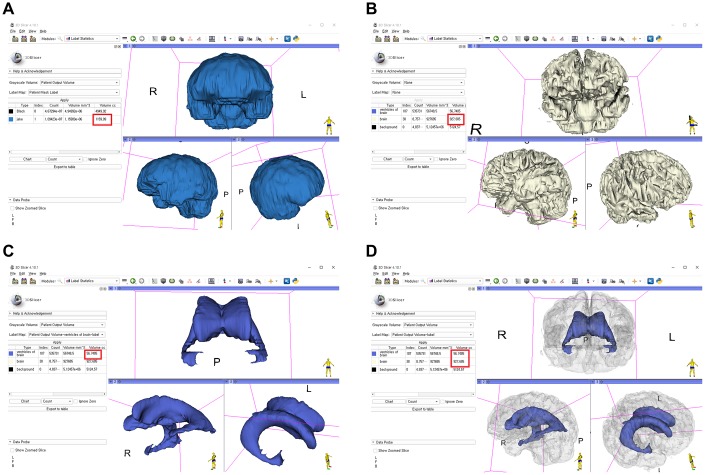
**Segmentation of the intracranial cavity, brain parenchyma, and lateral ventricles with a 3D-reconstructed model using the 3D slicer and calculation of each volume (red box indicates the volume).** (**A**) Intracranial cavity; (**B**) brain parenchyma; (**C**) lateral ventricles; (**D**) merge of the brain parenchyma and lateral ventricles.

The stepwise methods of volumetric assessment using 3D slicer were as follows: (1) brain MRI DICOM files from the picture archiving and communication system (PACS) were loaded to the software; (2) Swiss Skull Stripper function was used to strip off the skull from the loaded MRI to segment intracranial cavity; (3) threshold-based methods were then used to segment the brain parenchyma and lateral ventricles; (4) 3D reconstruction was performed using the Model Maker function; and (5) Label Statistics function was used to calculate the ICV, BPV, and LVV (Figure 6). Further detailed techniques are presented in Supplementary Figures 5–7.

### Medical variables

All medical information of the enrolled subjects was investigated by a trained research member using all medical charts, including nurse records. Age was defined as the age at the time of MRI. The individuals’ weight and height were investigated at or near the time of MRI from the medical records. BMI was calculated as weight/(height × height) and expressed in kg/m^2^. Medical history of hypertension, diabetes, alcohol drinking, and smoking at or near the time of MRI was also evaluated. A history of smoking and drinking was defined to include current smokers and drinkers (but not former smokers and drinkers).

### Statistical methods

Continuous variables are expressed as mean ± SD or median with interquartile range, while discrete variables are expressed as a count and percentage. The chi-square test and Student’s *t*-test were used to assess clinical differences between two groups. Statistical comparison between age groups was performed using one-way ANOVA followed by Tukey’s test (Tables 2 and 6).

Pearson correlation coefficients with/without adjustment for age were calculated to evaluate the relationships between volume percentage of brain parenchyma and lateral ventricles and bone densitometric parameters.

The intracranial cavity volume percentages of brain parenchyma and lateral ventricles were calculated as (BPV/ICV) × 100% and (LVV/ICV) × 100%, respectively. We constructed scatterplots with a regression line to represent the association between T-scores and the intracranial cavity volume percentages of brain parenchyma and lateral ventricles. Multivariable linear regression was also performed to identify independent associations between T-scores and the intracranial cavity volume percentages of the brain parenchyma and lateral ventricles. In addition to the T-score, covariates, including sex, age at the time of brain MRI (continuous variable), time interval between brain MRI and DXA (continuous variable), BMI (continuous variable), hypertension, diabetes, alcohol, and smoking were entered into the multivariable model.

Box plots with dot plots were used to visualize the association between the intracranial cavity volume percentages of brain parenchyma and lateral ventricles, and age groups according to with or without osteoporosis. P-values less than 0.05 were considered statistically significant.

All statistical analyses were performed using R version 3.5.2 (https://www.r-project.org/).

## Supplementary Material

Supplementary Figures
