# Supplementary material for: Association between bone mineral density and brain parenchymal atrophy and ventricular enlargement in healthy individuals
Source: Aging (Albany NY). 2019 Sep 30;11(19):8217–38. doi: 10.18632/aging.102316 (PMC6814624; doi:10.18632/aging.102316)
Supplement: Supplementary Figures [file aging-11-102316-s001.pdf]

SUPPLEMENTARY FIGURES

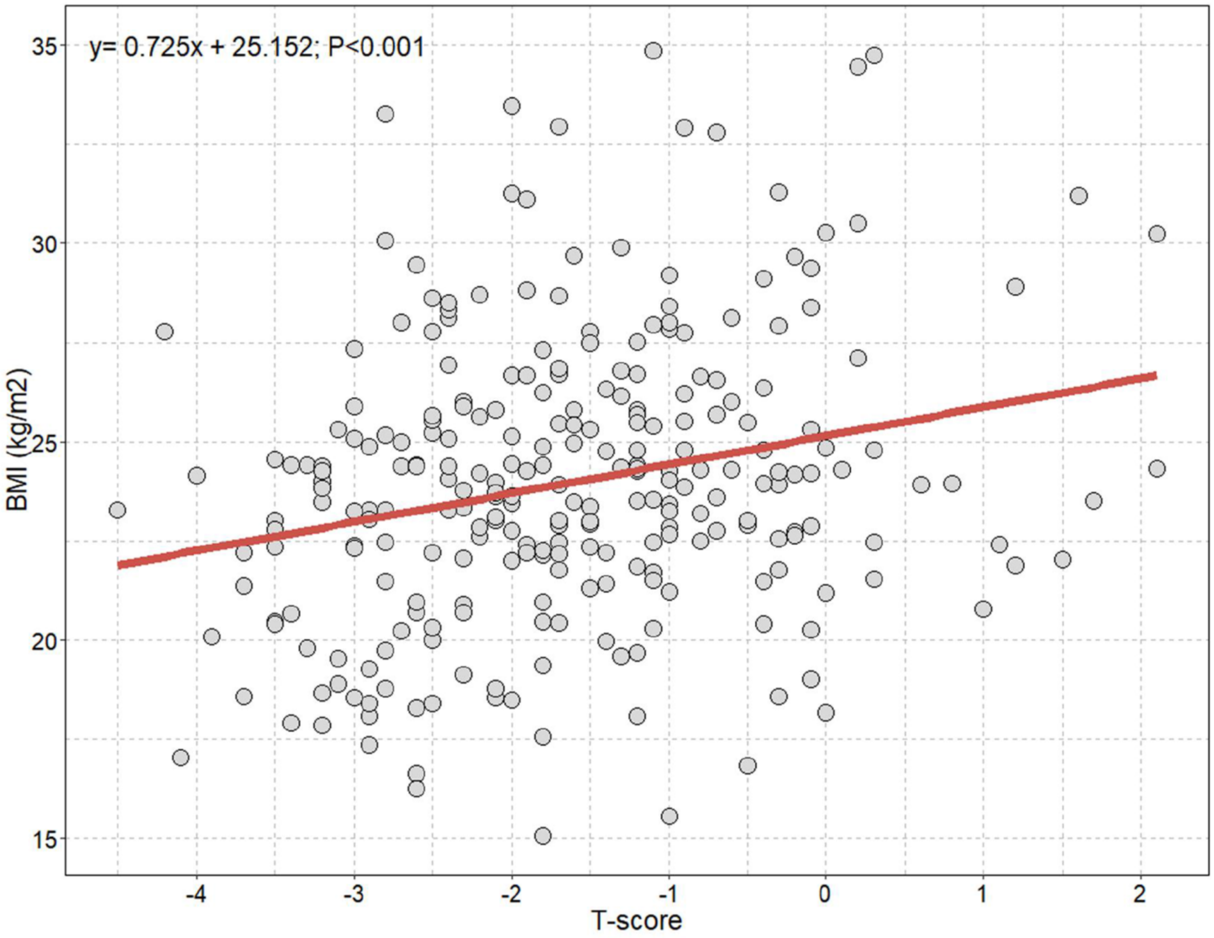

Supplementary Figure 1. Scatter plot with the linear regression line showing the association between T-score and BMI in the study individuals.

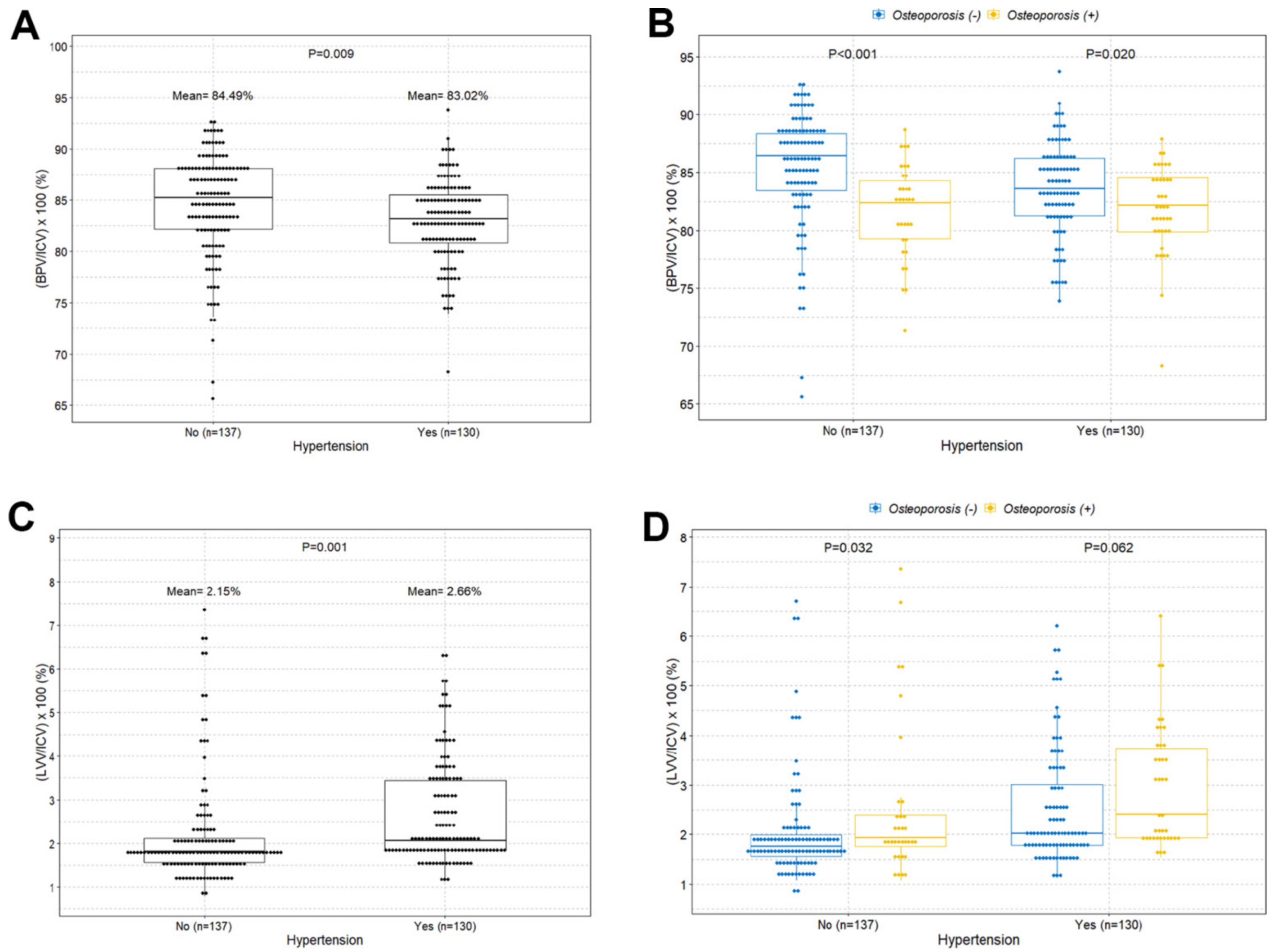

Supplementary Figure 2. Boxplots with dot plots of the volume percentages of brain parenchyma and lateral ventricles classified by history of hypertension. (**A**) volume percentage of brain parenchyma to intracranial cavity; (**B**) volume percentage of brain parenchyma to intracranial cavity according to osteoporosis; (**C**) volume percentage of lateral ventricles to intracranial cavity; (**D**) volume percentage of lateral ventricles to intracranial cavity according to osteoporosis. ICV=intracranial cavity volume; BPV=brain parenchymal volume; LVV=lateral ventricles volume.

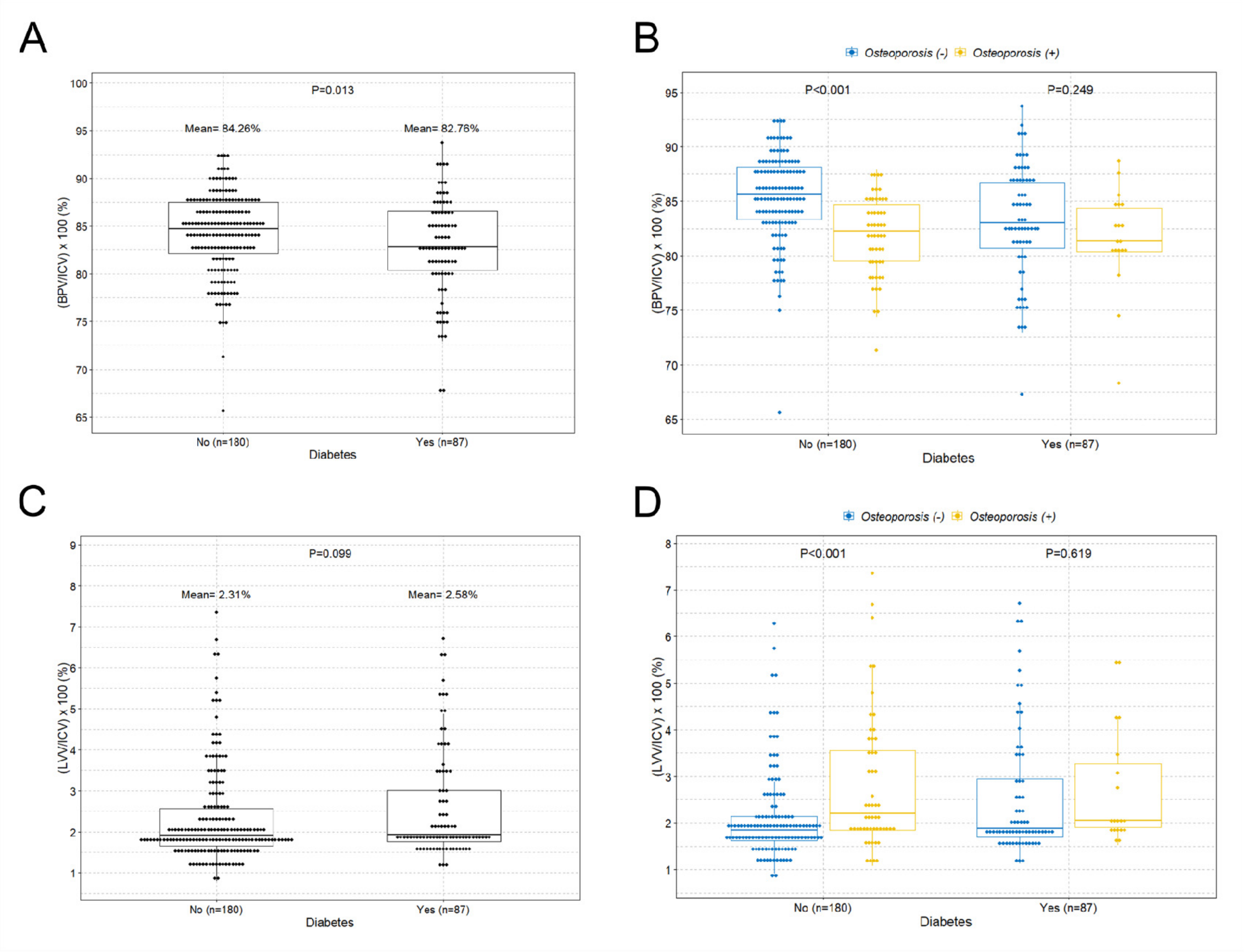

Supplementary Figure 3. Boxplots with dot plots of the volume percentages of brain parenchyma and lateral ventricles classified by history of diabetes. (**A**) volume percentage of brain parenchyma to intracranial cavity; (**B**) volume percentage of brain parenchyma to intracranial cavity according to osteoporosis; (**C**) volume percentage of lateral ventricles to intracranial cavity; (**D**) volume percentage of lateral ventricles to intracranial cavity according to osteoporosis. ICV=intracranial cavity volume; BPV=brain parenchymal volume; LVV=lateral ventricles volume.

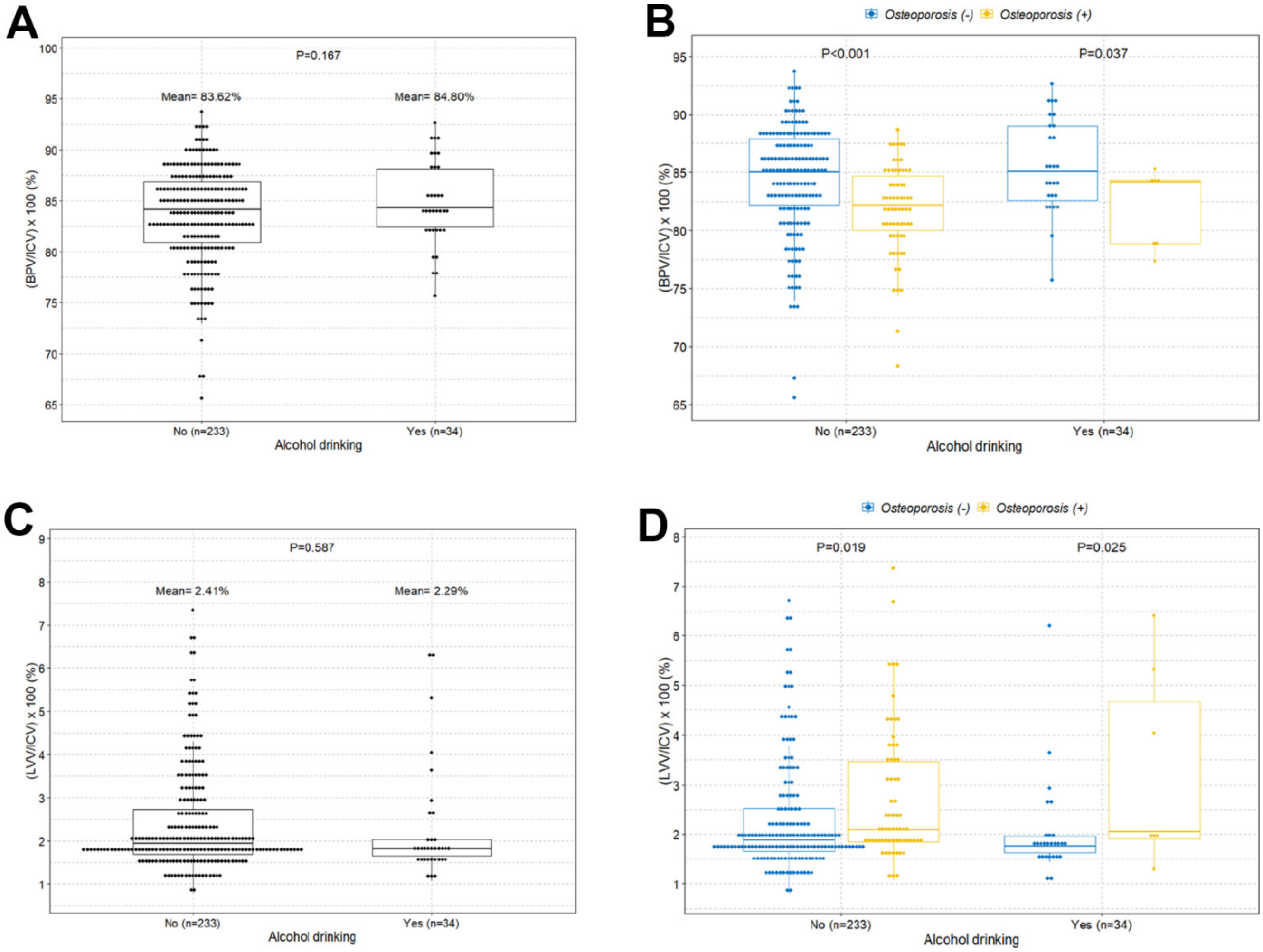

Supplementary Figure 4. Boxplots with dot plots of the volume percentages of brain parenchyma and lateral ventricles classified by history of alcohol. (**A**) volume percentage of brain parenchyma to intracranial cavity; (**B**) volume percentage of brain parenchyma to intracranial cavity according to osteoporosis; (**C**) volume percentage of lateral ventricles to intracranial cavity; (**D**) volume percentage of lateral ventricles to intracranial cavity according to osteoporosis. ICV=intracranial cavity volume; BPV=brain parenchymal volume; LVV=lateral ventricles volume.

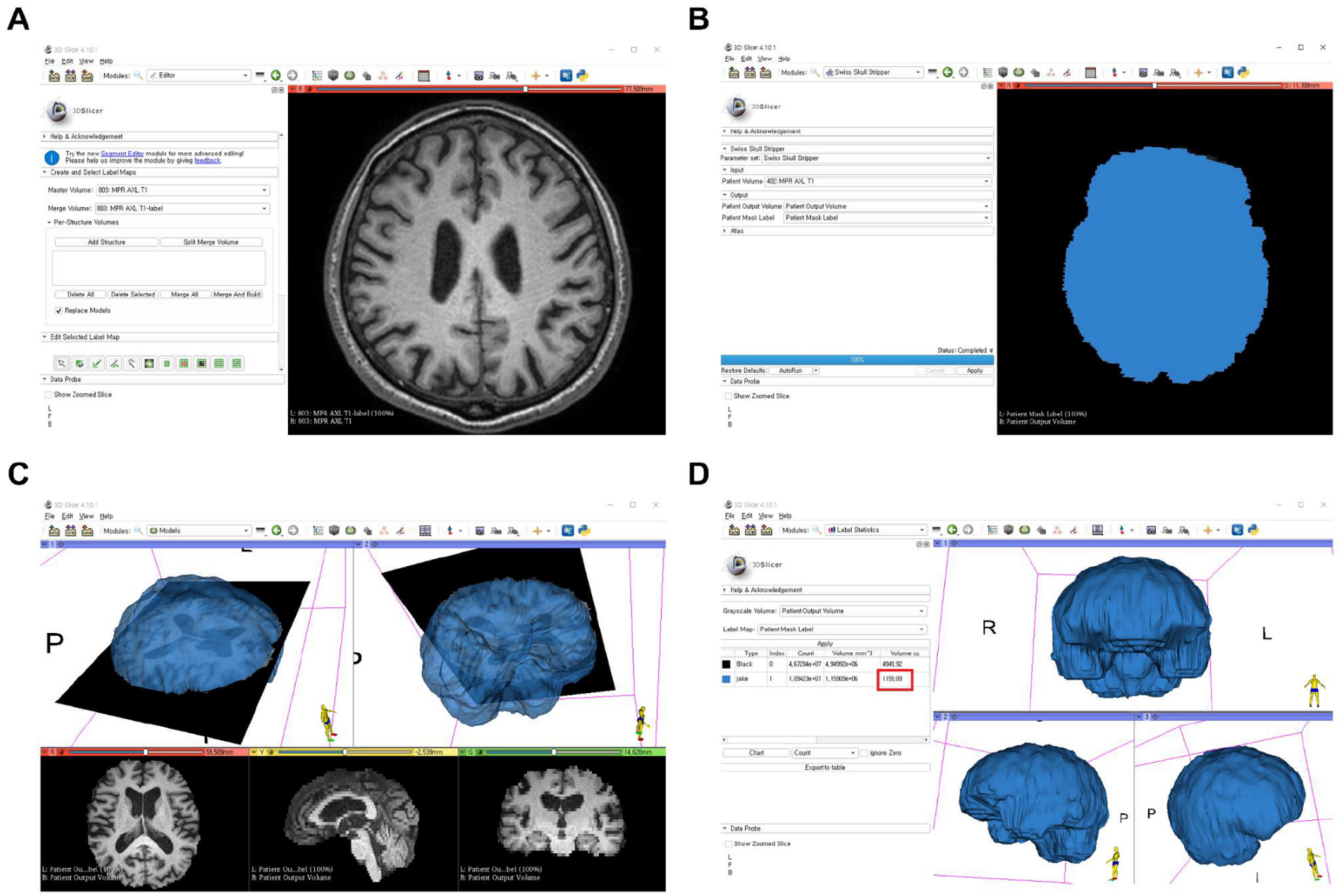

Supplementary Figure 5. Example of stepwise intracranial cavity volumetric assessment using 3D slicer. (**A**) brain MRI DICOM files from picture archiving and communication system (PACS) loading to the software; (**B**) Swiss Skull Stripper method used to segment the intracranial cavity; (**C**) 3D reconstruction was performed using the Model Maker function of the 3D slicer (**D**) Label Statistics function calculated the 3D reconstructed intracranial cavity volume.

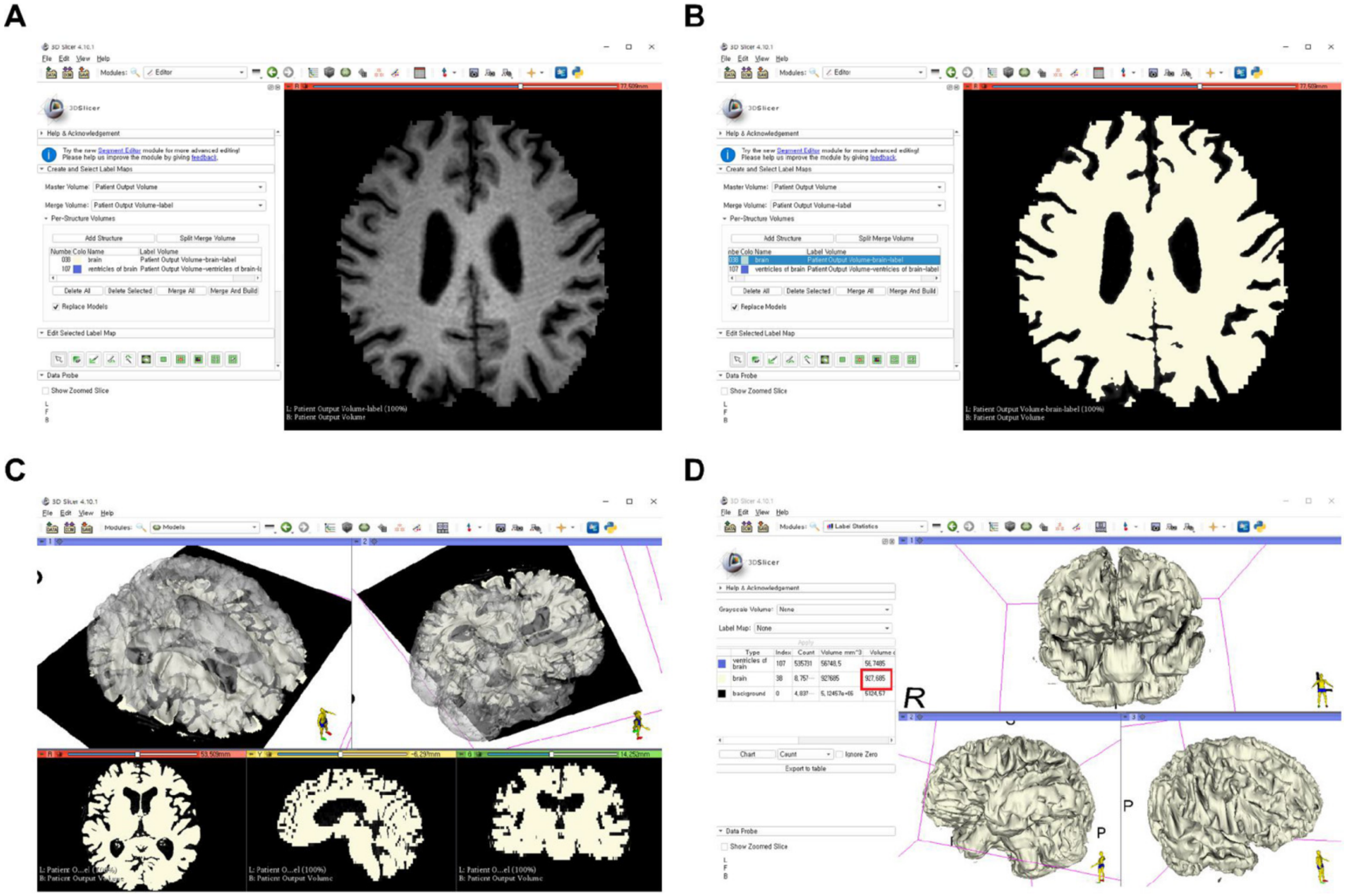

Supplementary Figure 6. Example of stepwise brain parenchyma volumetric assessment using 3D slicer. (**A**) brain MRI DICOM files from picture archiving and communication system (PACS) loading to the software. Swiss Skull Stripper method was then used to segment the intracranial cavity; (**B**) threshold-based method used to segment the brain parenchyma; (**C**) 3D reconstruction was performed using the Model Maker function of the 3D slicer (**D**) Label Statistics function calculated the 3D reconstructed brain parenchymal volume.

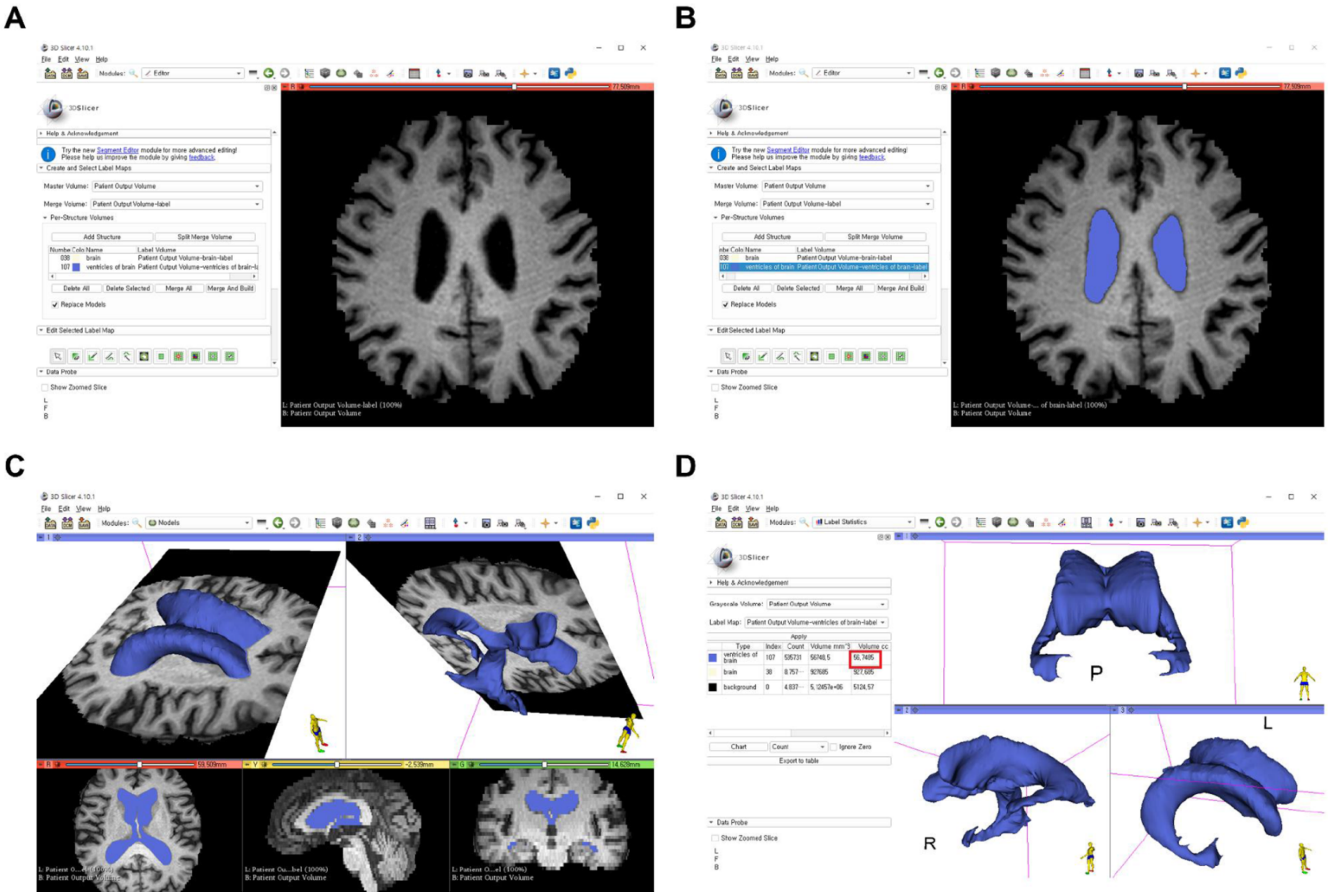

Supplementary Figure 7. Example of stepwise lateral ventricles volumetric assessment using 3D slicer. (**A**) brain MRI DICOM files from picture archiving and communication system (PACS) loading to the software. Swiss Skull Stripper method was then used to segment the intracranial cavity; (**B**) threshold-based method and Save Island function used to segment the lateral ventricles; (**C**) 3D reconstruction was performed using the Model Maker function of the 3D slicer (**D**) Label Statistics function calculated the 3D reconstructed lateral ventricles volume.
